# Outcomes important to patients with non-infectious posterior segment-involving uveitis: a qualitative study

**DOI:** 10.1136/bmjophth-2020-000481

**Published:** 2020-07-21

**Authors:** Mohammad O Tallouzi, David J Moore, Nicholas Bucknall, Philip I Murray, Melanie J Calvert, Alastair K Denniston, Jonathan M Mathers

**Affiliations:** 1 Institute of Applied Health Research, University of Birmingham, Birmingham, West Midlands, UK; 2 Birmingham and Midland Eye Centre, Sandwell and West Birmingham Hospitals NHS Trust, Birmingham, West Midlands, UK; 3 Patient Involvement Group in Uveitis (PInGU), Birmingham, West Midlands, UK; 4 Academic Unit of Ophthalmology, Institute of Inflammation and Ageing, University of Birmingham College of Medical and Dental Sciences, Birmingham, West Midlands, UK; 5 NIHR Birmingham Biomedical Research Centre, NIHR Surgical Reconstruction and Microbiology Research Centre and NIHR Applied Research Collaboration (ARC) West Midlands at the University Hospitals Birmingham NHS Foundation Trust and the University of Birmingham, Birmingham, West Midlands, UK; 6 Ophthalmology Department, University Hospitals Birmingham NHS Foundation Trust, Birmingham, West Midlands, UK

**Keywords:** inflammation, public health, treatment other

## Abstract

**Objective:**

Uveitis, a group of disorders characterised by intraocular inflammation, causes 10%–15% of total blindness in the developed world. The most sight-threatening forms of non-infectious uveitis are those affecting the posterior segment of the eye, collectively known as posterior segment-involving uveitis (PSIU). Numerous different clinical outcomes have been used in trials evaluating treatments for PSIU, but these may not represent patients’ and carers’ concerns. Therefore, the aims of this study were to understand the impact of PSIU on adult patients’ and carers’ lives and to explore what outcomes of treatment are important to them.

**Methods and Analysis:**

Four focus group discussions were undertaken to understand the perspectives of adult patients (=18) and carers (10) with PSIU. Participants were grouped according to whether or not their uveitis was complicated by the sight-threatening condition uveitic macular oedema. Discussions were audio-recorded, transcribed and analysed using the framework analytical approach. Outcomes were identified and grouped into outcome domains.

**Results:**

Eleven core domains were identified as important to patients and carers undergoing treatment for PSIU, comprising (1) visual function, (2) symptoms, (3) functional ability, (4) impact on relationships, (5) financial impact, (6) psychological morbidity and emotional well-being, (7) psychosocial adjustment to uveitis, (8) doctor/patient/interprofessional relationships and access to healthcare, (9) treatment burden, (10) treatment side effects, and (11) disease control.

**Conclusion:**

The domains identified represent patients’ and carers’ experience and perspectives and can be used to reflect on outcomes assessed in PSIU. They will directly inform the development of a core outcome set for PSIU clinical trials.

**Key messages**

**What is already known about this subject?**

- To date, patients’ and carers’ experiences of posterior segment-involving uveitis (PSIU) have not been explored in order to define patient-centred outcome domains that might be considered for use in clinical trials.

**What are the new findings?**

- This study publishes a qualitative piece of research informed by collected data from focus group discussions, proposing a novel outcome domain structure consisting of 11 core domains.

**How might these results change the focus of research or clinical practice?**

- These results provide the basis to reflect on the patient-centred nature of outcomes used in clinical research focused on patients with PSIU.
- The findings from this qualitative research study have been fed directly into the development process of a core outcome set for PSIU.

## Introduction

Uveitis describes a group of disorders characterised by intraocular inflammation responsible for 10%–15% of total blindness in the developed world and up to 25% of blindness in the developing world.^1–5^ Uveitis may be due to (1) an infectious agent or (2) non-infectious inflammation, either as part of an underlying systemic disease or as an isolated ocular phenomenon.^6^ Although uveitis may affect any age group, it peaks in the working-age population and has a disproportionately high impact in terms of years of potential vision loss,^7^ need for long-term therapy and socioeconomic impact.^8^

The most sight-threatening forms of uveitis are those that affect the posterior structures of the eye, classified anatomically as intermediate, posterior and panuveitis.^9 10^ In clinical trials, these forms of uveitis are often grouped together as non-infectious posterior segment-involving uveitis (PSIU), sharing a number of features including a higher risk of sight-threatening complications and often requiring systemic or local injection-based therapy. Uveitic macular oedema (UMO) is one of the common major complications in PSIU affecting around one-third of patients with uveitis.^7 11–13^ UMO is a leading cause of sight loss in patients since it affects the macula, which is responsible for detailed central vision. It, therefore, impacts a range of day-to-day activities such as reading, driving and working.

There is currently a major unmet need in the treatment of PSIU with a paucity of high-level evidence to allow evaluation and licensing of therapies by regulatory authorities^14^ and to inform treatment decisions by clinical experts and patients.^15^ The selection of outcome measures in trials is a critical issue, with evidence suggesting heterogeneity of outcomes used in recent clinical trials of uveitis,^14^ meaning that the outcomes from different trials may not be comparable. Further, patients, carers and health professionals may differ as to which outcomes they deem most important, and there may be a tendency for clinicians to undervalue outcomes that matter to patients.^16 17^ Consequently, these outcomes may not be included in uveitis research studies.

By understanding which outcomes matter to patients with uveitis and their carers, these perspectives can be included in the measured outcomes of uveitis trials to ensure that such trials are aligned to their priorities. These can be incorporated alongside the priorities of other stakeholders (clinicians, trialists, regulators and so on) into the construction of a core outcome set (COS) for uveitis, a recommended minimum set of outcomes to be assessed in all trials within the relevant health condition or group of conditions.^18 19^

COS methodology is well established and uses consensus methods with clinicians, patients, carers and other stakeholders to arrive at a recommended final set of outcomes.^20^ Qualitative research approaches can be used in the early stages of COS development to provide indepth understanding of the range of outcomes that have significance to patients and carers.^18^ As part of our study, ‘Development of a Core Outcome Set for Clinical Trials in non-infectious Posterior Segment-Involving Uveitis (COSUMO)’, qualitative research with adult patients and their carers was conducted to discover which outcomes matter to adult patients with PSIU, with and without UMO, and their carers.

## Methods

### Study design

This is a qualitative research study.

### Sampling

#### Eligibility criteria

Patient participants were eligible if they had a confirmed diagnosis of PSIU with or without UMO, for which they were receiving follow-up for uveitis. Participants were 18 or over with good spoken English and the capacity to give informed consent. For inclusion as a carer the participant had to be 18 or over and a friend, family member or spouse providing unpaid informal care to the patient during his/her illness and treatment journey.

#### Sampling characteristics

Sampling was undertaken with purposive criteria including patients of varying age, ethnicity and gender, with and without UMO.

#### Recruitment

Patients were recruited via the National Health Service (NHS, UK) uveitis clinics at the Birmingham and Midland Eye Centre (Sandwell and West Birmingham Hospitals NHS Trust) and University Hospitals Birmingham NHS Foundation Trust. Eligible participants were identified by consultant ophthalmologists and provided with an invitation letter and participant information sheet. A clinical research fellow (MOT) telephoned potential participants who had expressed their interests in the research, provided further information as required and answered any questions that arose. A suitable date, time and venue for a focus group discussion were arranged with those who agreed to participate. Written informed consent was taken from all participants on the day of the focus group prior to commencement.

### Data collection

Focus groups are a qualitative data collection method that is able to provide in-depth understanding of participants’ perspectives through group discussion and interaction.^21^ Patient participants were allocated to either a PSIU with UMO group (n=2 groups) or a PSIU without UMO group (n=2 groups), based on whether they had UMO within 2 years preceding the data collection. Carers attended the same focus group as the patients they were carers for. Four focus groups ran between November 2017 and February 2018.

Prior to the start of the discussion, participants completed a short background questionnaire in order to gather participants’ sociodemographic and clinical details. These data allowed monitoring and description of the sample characteristics. To supplement this, additional clinical data were collected from medical records by the clinical research fellow (MOT) with the consent of participants.

Private meeting rooms away from clinical areas were used to host discussions at the recruiting clinical sites. Audio-recorded discussions lasted between 87 and 106 min and were facilitated by the clinical research fellow (MOT) with the support of an experienced non-clinical qualitative researcher (JMM). A topic guide was designed to facilitate discussion. This was refined iteratively during the first two focus groups. Following an icebreaker discussion, where participants introduced themselves and gave a brief description of their uveitis background, three topics were used to provide data pertaining to participants’ perspectives on core outcomes from PSIU. These three topic areas were (1) the impact of uveitis on patients’ and carers’ lives; (2) the meaning of stable versus unstable disease; and (3) participants’ hopes and expectations related to treatment. The facilitators encouraged discussion and interaction, prompted to ensure that different group members were able to participate, and summarised discussion to probe for further insights around each topic area.

### Data analysis

A thematic analysis of content was informed by the framework analytical approach.^22^ Audio recordings were transcribed for analysis that was supported by the qualitative data analysis software NVivo V.12 (QSR International). Following initial data familiarisation, using both audio recordings and transcripts, a coding framework was developed iteratively by two researchers (MOT and JMM) in consultation with the broader research team. The focus group data were coded for outcomes. During this process our definition of an outcome was broad, including any consequence of PSIU or its treatment that clearly had significance to patients and carers who participated in the focus groups. Once we had finalised our coding framework, it was then applied to the whole data set (indexing). Data were then summarised descriptively, retaining the original meaning of participants’ discussions. Associative analysis was considered to assess similarities and differences between groups (eg, UMO vs non-UMO). Findings were discussed among the research team and at a COSUMO study meeting, where ophthalmologists, researchers and patient representatives were present. No significant changes to our analysis were necessary following these discussions. No novel issues emerged for discussion at the final focus group meeting.

### Patient involvement

A patient representative (NB) was involved in the study from the outset, was a coapplicant on the funding application and was on the steering group of the whole project. Specific contributions included providing advice on key aspects of the study design, including helping define the research questions, informing the constitution of focus groups and specifying the project outcomes. In addition they cowrote the patient information sheet. Patient groups (such as Birdshot Uveitis Society, Olivia’s Vision, Patient Involvement Group in Uveitis (West Midlands)) were also consulted to support the dissemination of this research.

## Results

A total of 47 participants were approached, and of these 30 agreed (64%) to take part and 17 declined (36%). Of the 30 who agreed, a total of 28 participants took part in the four focus groups: 18 patients and 10 carers, aged 31–72 years old. The sample included 4 male and 14 female patients, and 6 male and 4 female carers. Carers identified their relationships with the patients as spouse/partner (n=7), mother (n=2) and daughter (n=1). Uveitis was classified anatomically as intermediate (n=8), posterior (n=3) and panuveitis (n=7). Focus groups included UMO participants (n=7) and non-UMO participants (n=11). The main cause of uveitis was classified as idiopathic. A more detailed profile on the sociodemographic and clinical characteristics of the patients is shown in online supplementary tables 1 and 3, respectively. Carer characteristics are shown in online supplementary table 2.

10.1136/bmjophth-2020-000481.supp1 Supplementary data

10.1136/bmjophth-2020-000481.supp2 Supplementary data

10.1136/bmjophth-2020-000481.supp3 Supplementary data

### Identification of outcome categories and core outcome domains

During analysis 11 overarching outcome domains that have relevance to patients and carers were defined: (1) visual function, (2) symptoms, (3) functional ability, (4) impact on relationships, (5) financial impact, (6) psychological morbidity and emotional well-being, (7) psychosocial adjustment to uveitis, (8) doctor/patient/interprofessional relationships and access to healthcare, (9) treatment burden, (10) treatment side effects, and (11) disease control.

Table 1 explains the outcome domains, their definitions and the items in each domain.

**Table 1:** Outcome domains

Number	Outcome domains	Definition of domain	Items in the domain
1	Visual function	The impact of PSIU on aspects of patients’ vision.	Distance vision; near vision; colour vision; peripheral vision; contrast sensitivity; depth perception.
2	Symptoms	Patients’ bodily experiences that result from PSIU.	An uncomfortable or painful eye; photosensitivity; redness; watery eyes; floaters; visual disturbance; distortion of vision; headache; fatigue.
3	Functional ability	The impact of PSIU on patients’ ability to perform, maintain or continue their day-to-day functions.	Work/employment (maintaining/adjustments); educational participation; driving; activities of daily living and self-care; participation in social and leisure activities.
4	Impact on relationships	The impact of PSIU on relationships with others.	Intrafamily and spousal relationships; friendships.
5	Financial impact	The financial impact of having PSIU.	Financial cost to patients, for example, due to impact on work or treatment-related costs.
6	Psychological morbidity and emotional well-being	Psychological and emotional morbidity that may occur in patients with PSIU.	Depression; anxiety and stress; emotional well-being.
7	Psychosocial adjustment to uveitis	How well people with uveitis adjust to life with the disease and how it influences self-image. This partly results from day-to-day interactions with others, for example, family, friends and other people.	Threats to psychosocial well-being; coping strategies; indicators of psychosocial adjustment (reworked sense of self, identity, sense of normality).
8	Doctor/patient/interprofessional relationships and access to healthcare	An effectiveness of doctor–patient communication and between healthcare professionals; the ability to access uveitis clinics and uveitis care facilities.	Clinician–patient relationships; interprofessional communication; shared decision-making; access to health services and psychotherapy.
9	Treatment burden	The work that people with uveitis need to do to care for their health and its effect on their life.	Feeling of overall treatment burden; number of hospital visits; amount of medication; adherence.
10	Treatment side effects	Undesired effects of the treatment.	Treatment side effects (ocular and systemic).
11	Disease control	Control of PSIU and its complications.	Inflammation; complications (including raised intraocular pressure; UMO and cataract).

PSIU, posterior segment-involving uveitis; UMO, uveitic macular oedema.

#### Outcome domain 1: visual function

Patients identified vision as one of the most important issues in PSIU and referred to the impact of different visual impairments on their everyday life (table 2). Participants discussed a wide range of challenges faced as a result of impairments to different aspects of visual function:

**Table 2:** Components of visual function and impact on everyday life

Components of visual function	Definition of outcome	Examples of the impact on everyday life discussed in the focus groups
Distance vision	A patient’s ability to see objects/people clearly from distance (beyond arm’s length).	Difficulties in seeing faces, number plates, road signs, cars (only see the headlights), reading the guide on the television.
Near vision	A patient’s ability to see near objects.	Difficulties in seeing the remote control, phone numbers, menus, coins, the writing on a bottle of the shampoo; missing certain parts of text when reading.
Colour vision	A patient’s ability to distinguish colours accurately.	Difficulties in differentiating colours (eg, blues and yellows) or choosing items of clothing that match.
Peripheral vision	A patient’s ability to see towards the edge of their vision.	Difficulties in seeing items in the periphery of their vision.
Contrast sensitivity	A patient’s ability to distinguish objects from the background.	Difficulties in dealing with different contrasts between light and dark, for example, having to wear sunglasses on a foggy day.
Depth perception	A patient’s ability to perceive the world in three dimensions.	Difficulties in judging distances (eg, estimating how far away or how high a step is).

Phone numbers are dreadful, I get dazzled in that one, I’m reliant on my left eye which is pretty rubbish, and so you almost have to freeze for a bit and then your vision will come back, which is not great. (FG2P7)

Display stuff I really miss, especially at Christmas when they put all the bottles in the display stand. You just have to be really aware of that especially on the side of aisles and stuff that you don’t skip the aisle round and clip whatever on there, because part of your vision is gone from you. (FG3P3)

As illustrated by these quotes visual function was linked to aspects of functional ability, including activities of daily living, work and the ability to drive. The perceived impact of PSIU on visual function varied in discussions, based on disease severity and presence of complications, for example, UMO and raised intraocular pressure. For example, this participant described the impact of an episode of UMO on vision and functional ability:

When I had macular oedema, it was like seeing a dark hole in the middle of my vision so completely block the middle anything, missing spots in the central vision and I could not drive or work. (FG1P3)

During the focus groups progressive deterioration in visual function and related functional ability was a topic of discussion. This was sometimes linked to a perceived lack of effective medication:

My expectation was that the medication would work, and I would recover sight lost. I’ve had uveitis now for about ten years, now to the point where I’ve virtually got very limited vision. I’ve got virtually no vision in my right eye, very blurred and distorted vision in my left eye. Up until about six months ago I could read. Now I’m actually struggling to read or go out, can’t really watch TV. (FG4P4)

#### Outcome domain 2: symptoms

Participants reported a wide range of eye-related bodily symptoms as a result of PSIU. These are detailed further in table 3, including examples of the descriptive terms that participants used when talking about symptoms. Visual disturbance, distortion of vision and floaters were mentioned in all groups. For example, visual disturbances were described as a ‘milky haze’ or ‘steam’ and the resolution of which was associated with disease remission:

**Table 3:** Components of patients’ symptoms

Components of symptoms	Definition of outcome	Descriptive terms used by focus group participants
Uncomfortable or painful eye	A person complains of eye pain that may be severe and seem sharp, aching or throbbing, or a person may feel only mild irritation of the eye surface or the sensation of a foreign object in the eye (foreign body sensation).	Feeling irritation; scraping sensation or sandpaper when closing eyes, or experience of sharp pain; stabbing pain, terrible pain.
Watery eye	A person experiences a watery or a runny eye (excess tears).	Feeling your eyes streaming; like you have been crying.
Redness	A person experiences a visible bloodshot or redness to the white part of the eye.	Experience of having red eyes, a layer of blood go across the eye, and then as that goes down to my eyes it’s almost like it’s bloodshot.
Photosensitivity	A person experiences light intolerance or the eye is oversensitive to light (eg, in sunlight, fluorescent light, headlights, street lights).	Feeling light sensitive as just can’t stand any light at all; sensitive eyes to sunlight, fluorescent light, headlights, yellow bright light in the street.
Floater	A person complains of seeing moving dark or grey spots, specks, strands or cobwebs.	Seeing floating things; blob; seeing like a fly in front of vision; and black circles or dots going round. I had floaters and the only way to explain it is like a cling film over my eye that’s creased.
Visual disturbance	A person complains of seeing blurred, hazy, foggy, grainy vision, flashing/shimmering lights or double vision.	Seeing fog in front of vision; hazy vision; flashing lights; shimmering lights; a drifting across my eyes; grainy vision; loads of steam. Seeing things but not defined, but it’s just like a milky haze.
Distortion of vision	A person complains that straight lines may appear bent, crooked or wavy.	Seeing things wavy rather than straight and lines appear bent.
Headache	A person experiences a severe or throbbing headache.	Feeling headache I can’t spend more than an hour on the computer, because it just gives me bad headache; it is like throbbing.
Fatigue	A person experiences fatigue, exhaustion, feeling tired or lack of energy.	Feeling tired; very exhausted; I feel I am a sleepy person.

You can sort of see things but it’s nothing defined, but it’s just like this. I always think of it like a milky haze, you can also say it’s like opaque. Some days there’s not a lot of steam, some days there’s loads of steam. And you know when it’s getting better that steam starts to disperse, the lines are wiggly and they’re not straight, and I’m looking at that and a bit of that letter disappears. (FG2P5)

Another discussion in the first focus group highlighted patients’ experience of eye discomfort and pain, which for some was significant:

I think it’s because I’ve had it so long you just know it’s going to happen. You get a certain pain that it’s like almost a bit like a stabbing pain, terrible pain I know I’m in trouble. (FG1P3)

I actually do get quite a lot of irritation. I don’t get pain with it actually, so I get really dry irritated eye, like how can you put it? Like you’ve got something stuck in it? Gritty? Yeah like that. Like sandpaper? Yeah, something like that yeah, when you close your eyelid it scrapes. (FG1P4)

Additionally, fatigue was discussed as a significant issue by patients:

When I first started getting uveitis I was falling asleep at stupid times during the day. I would be talking to somebody one min, and that was when uveitis first started, and my ex-wife she says to me the one day, ‘Your eye is ever so red.’ I said, ‘I’m just tired, I’m really exhausted’. (FG3P3)

#### Outcome domain 3: functional ability

Patients and carers discussed how uveitis and related loss of vision impact people’s ability to function in a range of areas, including work, educational participation (eg, attending university), driving, activities of daily living and participating in social and leisure activities (table 1). For example, the impact on work, both the ability to maintain working life and adjustments required in work to do so, was mentioned frequently, as was the ability to drive. These contributed to maintaining a sense of independence and autonomy:

But also, it has impacted my life. I have to consider if I lost my sight how would I function. Because I live alone and when you live alone and to lose your independence it’s a major thing for me, and driving independence again, if you lost that I would be lost. Working, when I’m at work and I have to ask somebody, or I’m going through my bad period and got to be. I’m a tutor and I do a lot of paperwork, a lot of computer work, and I just find it a struggle all the time, and then that has a physical impact, and I just feel stressed and thinking oh my God am I going to lose my sight? (FG2P2)

Impacts on activities of daily living and the ability to self-care were also key concerns for both patients and carers. Here one participant talked about uveitis impacting their ability to shave:

I asked her, ‘Can you shave me?’ She goes, ‘I can’t shave you, I can’t.’ So, I go in the bathroom and because I can’t see my face I can’t see where I’m going so I’ve got too much off that and start bleeding everywhere on my face. (FG4P3)

Discussion also covered participation in social life and leisure activities:

For him who would normally go out with his cousins and he can’t go out with them because his vision is not good. He used to love going to the gym. He can’t go to the gym. So, his activities are what he used to do for a social point of view have all changed. (FG2PC5)

#### Outcome domain 4: impact on relationships

Participants discussed the impact of PSIU and subsequent treatment on intrafamily and spousal relationships and future family unity. During one discussion a father with PSIU and his daughter reflected on the effect that the disease had on their family unit, relating this to a lack of understanding of the disease and its impacts on the part of the father’s spouse:

It’s impact on the family unit as well. Yeah, the amount of arguments we’ve had because of it, and it isn’t like arguments because we’re upset with you that you have it, it’s just I think again my mum she wants to understand it and she tries to understand it, but then I have to pull her back a bit and you’ve got to realize what you’re saying because this is not his fault. (FG1PC1)

Other issues discussed included the impact of treatment on the ability to have children and on the relationship between parents and their children. The impact of PSIU was not limited to family relationships; it was also discussed in the context of friends, where participants had felt the need to distance themselves from friendships and colleagues because of a lack of understanding of PSIU on the part of others:

People at work, friends, a lot of people I have had to distance myself from, because of the prejudice, because they don’t understand what it’s like to have something that is basically immune system eating your eyes which is essentially what this disease is. (FG2P7)

#### Outcome domain 5: financial impact

The financial impact of uveitis was discussed in detail and perceived as an important outcome among participants. The main source of this was related to early retirement or redundancy due to loss of vision and being unable to work. However, the additional financial burden associated with travel to specialist eye services and the cost of treatments was also highlighted.

#### Outcome domain 6: psychological morbidity and emotional well-being

The psychological and emotional impacts of PSIU were discussed in all of the focus groups and they were frequently raised by patients and family members, especially carers. Depression was a significant issue as illustrated by the following quote:

I had three injections of steroids over the course of a week, and I was euphoric to start off with, and I wanted to die. I was so happy I wanted to die, seemed like a perfect time to die, and I was so mad I didn’t know how to. I just thought all I have to do is say bye bye world and that’s it I’m gone. And then the depression set in, and I am not one of these depressed people who goes to bed and pulls the duvet over me, but felt raging and aggressive and very unpleasant to be around. (FG2PC1)

Anxiety and stress were also raised. Influences on this were suggested to include the fear of sight loss, uncertainty and stress concerning the occurrence of flare-ups and the effectiveness of treatment, and anxiety about what the future holds. Emotional well-being, including feelings of frustration and anger, for example, when patients were in pain, was also a component of this domain.

#### Outcome domain 7: psychosocial adjustment to uveitis

While psychosocial adjustment will be influenced by psychological morbidity and emotional well-being, this domain is distinct and describes how well patients with PSIU are able to adjust to life with the disease and how it affects their self-image. This is influenced by day-to-day interactions with others. We have defined three components to this domain: (1) threats to psychosocial well-being—the things that indicate that patients are having difficulties with adjustment; (2) coping strategies—the strategies that people adopt in order to master, tolerate or reduce the impacts of PSIU on psychosocial issues; and (3) indicators of psychosocial adjustment (table 4).

**Table 4:** Components of psychosocial adjustment to uveitis

Components of psychosocial adjustment to uveitis	Definition	Examples
Threats to psychosocial well-being	Things that indicate that individuals are having difficulty with psychosocial adjustment to uveitis or going through a process of adjustment, for example, social anxiety, acceptance of the disease, social reaction, changing personal items, autonomy and independence.	Grief for losses incurred, for example, vision/sight loss. Lack of acceptance of the disease and adjustments to life required. Lack of predictability of the disease and impacts-related uncertainty regarding the future. Anxiety related to perceived or actual social reactions to the person with the disease. Feeling of dependence on others and loss of autonomy. Sense of role disruption, for example, work, family. Need to change things that are components of self-image, for example, unable to wear make-up, unable to wear items of clothing that are tied to self-image.
Coping strategies	Things that individuals with uveitis do in order to cope with threats to their well-being and psychosocial adjustment. These can include a mix of psychological and behavioural strategies and can have an effect on how well people with uveitis are able to adjust.	Psychological, for example, acceptance of disease and impacts, adopting a positive attitude, good mindset, positive spiritual beliefs. Behavioural strategies and modifications, for example, changing driving behaviour, changing work or adjustments at work, change in day-to-day life patterns. Self-management, for example, pharmacological and non-pharmacological interventions that would help to prevent the disease from worsening, such as increasing eye-drops, lifestyle modifications such as diet, relaxation techniques.
Indicators of psychosocial adjustment	Indicators that people with uveitis have gone through processes of psychosocial adjustment.	Sense of self: how people with uveitis view themselves, that is, their self-image. Identity: how people with uveitis are viewed by and relate to others. Sense of normality: a person’s ability to retain or regain some sense of normality within life.

Various threats to psychosocial well-being were discussed in the focus groups, for example, anxiety related to social reactions towards the person with uveitis, or a feeling of loss of autonomy and independence:

I don’t like crowds really, and I’m always conscious if anybody is next to me. Because you can’t see out of your eye, and you don’t know if you’re walking into anybody, so normally I grab onto someone’s arm or something, just so you don’t make a fool of yourself and walk into things. I think it’s made you quite…I’m conscious that it’s made [referring to participant’s husband] quite hyper-vigilant of me, I’m quite panicking in some ways; When we’re coming up to a road I’ve been very conscious that you grab hold of my hand, because you’re thinking oh am I going to bump into anyone. (FG1P4)

In response to these threats, participants talked about a range of coping mechanisms. These can include a mix of psychological (eg, acceptance of PSIU, positive attitudes, spiritual beliefs) and behavioural strategies (eg, changing driving, reading or working behaviours or change in day-to-day routines):

There was a point where for days I couldn’t get out of bed, what’s the point? And then I’m thinking I’m lying in bed wasting these days, get up and do something. That’s what I try to do, what you should do… (FG1P2)

So, I suppose in some ways what I found the fact that I’ve got used to uveitis. I found ways of working round it, and I’ve got the flexibility that I can do. But obviously I was lucky that took place, so yeah. (FG3P1)

There may be certain indicators that individuals have gone through these processes of psychosocial impact and adjustment, successfully or not. Over time this may be a cyclical process dependent on disease progression and the impacts related to this. From the focus group discussions, we have defined three concepts that signify psychosocial adjustment or a lack of it: sense of self, sense of normality and identity.

Sense of self describes how patients with uveitis view themselves, for example, their self-image. It is influenced by interactions with others. This could be positive or negative or mixed, for example, feeling productive, useful, guilty, helpless or useless. Uveitis threatens sense of self:

For me it’s about self-image and self-body image to start with. But that’s the one that feeds into the having to come to terms with your different abilities, and your different image and therefore the view that what others may think of us or think of me. (FG3P1)

In describing the impact of PSIU on sense of self, this participant also touches on impacts on identity, for example, how individuals with PSIU relate to others in their social and professional networks. Participants also described a desire to ‘go back to normal’ and to regain a sense of normality in life:

The aim was to maintain his [the participant’s son] normality. So, he wants to be independent despite all what’s going on with him. (FG2C4).

What’s normal for me is when I can function and when can I start and complete a task, and when I’m not really on medication well it’s being able to function normally and do your everyday stuff. (FG3P3)

Each of these concepts, a reworked sense of self with uveitis, identity or a new sense of normality, may be indicators of psychosocial adjustment.

#### Outcome domain 8: doctor/patient/interprofessional relationships and access to healthcare

This domain includes the quality of relationships between clinicians and patients; interprofessional relationships and communication between clinicians; and access to services (ie, eye, social and psychological). Often these were interrelated, with patients’ perceived quality of relationships with clinicians being influenced by access:

The clinic was so busy, and Mr. M was a superstar and squeezed me in literally, I rang the one day, I was in the next day, but the clinic was heaving, you don’t like to bother them either. May be that’s where I’m wrong. (FG1P2)

One of the first things he said to me was that if anything changed with my sight I had to ring him, and my previous experience at the local eye clinic I lost all sight in one eye, I rang the eye clinic, emergency eye clinic to get an appointment; nobody answered the phone for an entire day. (FG1P1)

Participants identified a significant deficit in access to psychotherapy and counselling services, sometimes comparing this with other disease areas:

It’s really hard to get emotional support, because you do need emotional support, it’s like you said there is no psychological counselling. I waited nine months to get NHS counselling from my GP, but it’s only your eyes and you’re getting all your medication so you must be fine living with it day to day, and you’re getting all these injections. But it doesn’t consider the psychological impact, the anxiety. You just don’t know, I don’t know if I’m going to be able to see my daughter’s face the next day or watch her do her school plays or things like that, and causes an immense amount of anxiety for me. But there’s no helpline, there’s nothing. (FG2P3)

#### Outcome domain 9: treatment burden

Treatment burden is the workload of healthcare undertaken by patients and carers. Participants described feeling a significant burden of care, for example, associated with the number of follow-up appointments, waiting time, duration of treatment and the impact on family members who had to give up work to care for their partners. Further, the treatment burden in uveitis comprises the work of developing an understanding of the condition and treatments, interacting with others to get clinic appointments, taking medications and enhancing lifestyle factors. It was linked to participants’ engagement in activities such as work, studying, leisure and being with friends and family, and influenced psychosocial adjustment:

I was having triamcinolone injections and Ozurdex implants literally every six weeks, and that was because I’ve been in a constant flare for three and a half years. So, it never went away, and then that caused problems with glaucoma and had the treatment for that. So I couldn’t work because I was constantly at hospital, and being treated constantly. So since February this year having the Iluvien allowed me to do more and go back to be normal, and go back to work, which I never thought I would ever say I would get excited about. (FG2P7)

Focus group participants also discussed the routines they had to establish around treatment and how burden and routine could influence treatment adherence:

You have to set your phone to put an alarm on it every few hours to do it. You have to put in practices and procedures to enable the treatment. Because treatment is fine if you comply with it, but if you don’t comply it’s no good. At the moment my eye drops are in the morning and of an evening so that’s not too bad, it’s just remembering, especially now that I’m working, remembering to take my tablets to work to take them at the right time. (FG1P4)

#### Outcome domain 10: treatment side effects

Most of the patients reported experiencing significant side effects from medication, particularly corticosteroids. These included ocular side effects, such as irritation and discomfort, and systemic effects, such as feeling generally unwell or weight gain. Participants linked side effects to negative psychological and psychosocial impact, identifying additional treatment burden imposed by medications prescribed to address side effects.

I had that emotional mood swings, I don’t know what the doctor said, but I basically got really bad oral thrush, and I couldn’t eat or drink for a week. (FG2P3)

#### Outcome domain 11: disease control

Uveitis by definition is an intraocular inflammation; therefore, control of inflammation and flares was perceived as an important outcome for patients with PSIU:

I had one treatment which I knew that you couldn’t have long term. Then my expectation from that treatment was that it would stop the inflammation and the high pressure at the same time. (FG2P3)

Patients experienced a number of ocular complications including raised intraocular pressure, macular oedema or cataract, and controlling those complications was perceived as being an important outcome. Further, when participants perceived their disease was under control, they were able to regain their functional ability and sustain their sense of normality:

I ended up with cataracts in both eyes, raised ocular pressure, glaucoma drops, and not able to even see my feet. Then I had surgery, vitrectomy and cataract surgery, sight came back, but medication I have been on before haven’t controlled the inflammation, didn’t control inflammation afterwards, and I ended up having Avastin and Lucentis injections, and they gave me the sight to be able to work again. (FG1P4)

## Discussion

The aim of this research was to identify outcomes and outcome domains that are important to adult patients with PSIU and their carers. To our knowledge, this is the first qualitative research study to do this. From our analysis of the qualitative data collected, we have proposed 11 domains that cover the issues that were clearly significant to participants in this research study.

The current paradigm for PSIU assessment is based on outcomes reported by the Standardization of Uveitis Nomenclature (SUN) workshop in 2005.^10^ Although this was a major step forward in the process of standardising methods for reporting outcomes and clinical data in the field of uveitis,^11 23^ it was based on the opinion of clinical experts, without patients’ or carers’ input. It focused on items that would all be categorised within the disease control and visual function domain described in this paper and does not reflect the broader set of issues discussed by patients and carers who attended the focus groups. Systematic reviews of trials in PSIU similarly demonstrate that clinical studies have predominantly focused on disease control and visual function outcomes,^14 24^ with a smaller number of trials looking at treatment side effects or quality of life.^23 25–27^

Disease control outcomes reported in clinical trials include inflammatory activity (eg, anterior chamber cells and vitreous haze), disease complications (eg, macular oedema, raised intraocular pressure and cataract) and visual function.^14 24^ Interestingly distance vision is the only measure of visual function used in clinical trials and has been a standard test in the clinical practice of ophthalmology.^14 24^ However, this qualitative work highlights that from a patient’s perspective the impact of PSIU on visual function is multifaceted and goes beyond what is being measured currently to include near vision, peripheral vision, colour vision, contrast sensitivity and depth perception. Additionally, focus group participants discussed ocular symptoms, such as visual disturbances and distorted vision, all things that were seen to impact on functional ability and other broader outcome domains identified here. Some participants reported good distance vision while experiencing ocular symptoms that impinged on the clarity of their vision. Other systemic symptoms were also discussed by patients and carers, with fatigue being mentioned most frequently.

Domains commonly discussed in the focus groups but not covered in SUN or clinical trials in uveitis were functional ability; the impact on relationships and finances; psychological morbidity and emotional well-being; psychosocial adjustment to uveitis; doctor/patient/interprofessional relationships and access to healthcare; and treatment burden. The impact on diverse functional abilities (including employment, activities of daily living and self-care, driving and participation in social and leisure activities) was a key consequence of disease activity, impairments to visual function, symptoms and the treatment burden experienced. In turn, this could impact on finances and relationships. Focus group participants discussed resultant psychological morbidity including depression and anxiety, and also talked about influences on day-to-day emotional well-being.^28 29^

While potentially influenced by psychological morbidity and emotional well-being, we have also identified a separate domain describing patients’ psychosocial adjustment to life with uveitis. We believe that this is an important domain to consider separately to the others presented here. It describes people’s adaptation to life with uveitis and how the disease and its consequences impact on self-image (people’s sense of self), something that has been identified as a fundamental concern for those living with debilitating chronic conditions.^30^ The importance and nature of adjustment to chronic disease have been studied in other conditions and theorised in both the sociology and psychology of medicine.^31–33^ From the focus group data, we were able to identify how participants talked about the things that indicate difficulties with psychosocial well-being, psychological and behavioural coping strategies that they adopted, and potential indicators of adjustment.

As demonstrated here there is obvious discordance between the diversity of outcomes currently assessed in uveitis research and practice, highlighting the range of concerns that patients and their carers report in this qualitative research study. The question remains as to the implications of this for future outcome assessment in research and clinical practice. One could argue that the broader impacts of PSIU described by patients and carers, for example, functional impairment and psychological and psychosocial impacts, are all sequelae to disease activity and visual function, and hence assessment of those outcomes, that is, the status quo, will implicitly reflect the broader impacts that patients and carers give weight to. However, in the absence of cure, or treatments that allow patients to live their lives as if there was an absence of disease (total and permanent remission), there remains a question regarding the association in response between measures of disease activity and visual function and the broader issues within patients’ lives that they discussed in the focus groups. In other words, do incremental improvements in treatment outcomes measured by disease activity and visual function translate to significant improvements in patients’ lives overall? Establishing this would seem crucial considering the impact of significant treatment burden that patients and carers described.

Traditionally this has been an argument for measures of health-related quality of life (HRQoL) to be incorporated in clinical research and practice. Within the domain framework we present here, we have not identified components of HRQoL or referred to this concept specifically. Patients and carers did not actually refer to quality of life as an umbrella term within the focus group discussions. There are often varied definitions and conceptualisations of HRQoL. Without detailed reflection on the components of measures, there is a danger that by including any measure of HRQoL we assume that patients’ and carers’ views are being incorporated in patient-centred manner. However, this may not necessarily be the case, for example, where psychosocial adjustment is not a component of HRQoL measures. This is becoming recognised, for example, with a recent call to incorporate adjustment into the International Classification of Functioning, Disability, and Health,^34^ and with work examining specific tools available to assess living with chronic conditions.^35^

It is worth noting that the National Eye Institute Visual Functioning Questionnaire-25 has been validated for many eye conditions, including macular degeneration, cataracts, diabetic disease and glaucoma.^29 36^ However, its validation among patients with uveitis is limited.^29 37^ Interestingly, this tool covers items related to several of the domains identified here, including aspects of functional ability, visual function, emotional well-being and certain threats to psychosocial adjustment.

### Strengths and limitations

This is the first qualitative research study to investigate patients’ and carers’ views regarding the outcome domains relevant to outcome assessment in PSIU. The focus groups were successfully conducted with a relatively diverse range of adult patients and their carers by researchers with a clinical background in PSIU and an experienced qualitative researcher. All four groups generated in-depth discussion of issues in a participant-led manner. Participants were keen to share their views with other attendees and researchers. No new issues were emerging for discussion by the fourth focus group, suggesting that our domain structure provides a comprehensive picture of the issues of importance to patients and their carers, and that saturation had been achieved.^38^

There are some limitations to our study. First, we did not include children and do not therefore claim that the outcome domains presented here will represent the experience of children or their guardians. Second, while grouping dyads of patients and carers together within the same focus groups was more practicable and allowed joint discussion of issues from a carer and patient perspective, we cannot be certain whether individual participants may have been inhibited in discussing certain issues in front of significant others. Finally, while purposive sampling was successful across a range of characteristics, we were unsuccessful in recruiting from certain ethnic groups despite recruiting from a strongly multiethnic clinical service; it is recognised that certain communities and ethnic groups are more hesitant to engage with research, and this is a recurrent challenge in such studies.^39 40^ Similarly, many studies including ours have noted the difficulties in recruiting the younger adult age group (18–30 years), usually attributed to work or family commitments at this stage in people’s lives.^41^

## Conclusion

We suggest that this outcome domain framework can be used as a basis for reflection on the outcomes assessed in research focused on adult patients with PSIU and their carers. Domains detailed here are not currently encompassed in trial research within PSIU. This work forms part of the basis for the COSUMO study (manuscript in preparation). The development of a COS for PSIU would provide for the first time a standardised set of outcomes that has value to all stakeholders and can be used in future effectiveness trials in uveitis. The value of COS is increasingly recognised. Benefits include maximising the value of each clinical trial since key outcomes are measured and reported in all relevant trials; ensuring that outcomes measured include those that are most important to each group of stakeholders, rather than just to one group; reducing outcome selection bias and outcome reporting bias since the whole COS is measured and reported; and improving the statistical power of any meta-analysis since more studies can be included.^18 42^ The outcome domains detailed in this research are highly relevant to a COS in uveitis, and this is a key step in the development process to ensure that such a COS includes the outcomes that matter most to patients with PSIU and their carers.
