# Supplementary material for: Outcomes important to patients with non-infectious posterior segment-involving uveitis: a qualitative study
Source: BMJ Open Ophthalmol. 2020 Jul 21;5(1):e000481. doi: 10.1136/bmjophth-2020-000481 (PMC7375431; doi:10.1136/bmjophth-2020-000481)
Supplement: Supplementary data [file bmjophth-2020-000481supp001.pdf]

Supplementary Table 1: Patient characteristics

Characteristic		N (%)
**Gender**	Male	4 (22)
	Female	14 (78)
**Age Range (yrs.)**	18-30	0 (0)
	31-45	4 (22)
	46-60	11 (61)
	Over 60	3 (17)
**Ethnic group**	White	16 (89)
	Mixed/ multiple ethnic groups	0 (0)
	Black/African/ Caribbean/ Black British	2 (11)
	Other	0 (0)
**Employment**	Employed	12 (67)
	Unemployed	1 (6)
	Housewife/househusband	0 (0)
	Retired	5 (28)
**Smoking**	Never smoked	7 (39)
	In the past	8 (44)
	Current smoker	3 (17)
**Driving**	Never driver	1 (6)
	Current driver	14 (78)
	In the past	3 (17)
