# Supplementary material for: Outcomes important to patients with non-infectious posterior segment-involving uveitis: a qualitative study
Source: BMJ Open Ophthalmol. 2020 Jul 21;5(1):e000481. doi: 10.1136/bmjophth-2020-000481 (PMC7375431; doi:10.1136/bmjophth-2020-000481)
Supplement: Supplementary data [file bmjophth-2020-000481supp002.pdf]

**Supplementary Table 1: Clinical characteristics of patients**

| **Anatomical type of uveitis** | **N (%)** |
|---|---|
| Intermediate | 8 (44) |
| Posterior | 3 (17) |
| Panuveitis | 7 (41) |
| **Aetiology/syndrome of uveitis** | |
| Idiopathic | 13 (72) |
| Punctate Inner Choroidopathy | 1 (6) |
| Multiple sclerosis- associated uveitis | 1 (6) |
| Birdshot chorioretinopathy | 2 (11) |
| Seronegative arthritis-associated | 1 (6) |
| **Duration of uveitis** | |
| 1- 5 years | 6 (33) |
| 6-10 years | 2 (11) |
| Over 10 years | 10 (56) |
| **Current uveitis activity status** | |
| Active in one eye | 5 (28) |
| Active in both eyes | 2 (11) |
| Inactive | 11 (61) |
| **Longest duration of reduced vision in affected eye (6/18 or worse) in the last 2 years** | |
| 1-3 months | 6 (33) |
| 4-8months | 4 (22) |
| 9-14months | 1 (6) |
| Over 15 months | 5 (28) |
| **UMO present in the last 2 years** | |
| Bilateral asynchronous | 3 (17) |
| Bilateral simultaneous | 6 (33) |
| Unilateral | 9 (50) |
| Bilateral | 9 (50) |
| **Number of macular oedema episodes in the last 2 years** | |
| 1 episode | 2 (11) |
| 2 episodes | 4 (22) |
| 3 episodes | 1 (6) |
| 4 episodes | 1 (6) |
| Never resolved | 1 (6%) |
| **Current macular oedema status** | |
| Present in one eye | 2 (11) |
| Present in both eyes | 0 (0) |
| Absent | 7 (41) |

| **Systemic treatment in the last 2 years** | | | **Local treatments in the last 2 years** | |
|---|---|---|---|---|
| Oral prednisolone | 0 | | Periocular corticosteroid | 1 (6) |
| IVMP | 2 (11%) | | Intravitreal Triamcinolone | 3 (17) |
| Deep IMMP | 1 (6%) | | Intravitreal Ozurdex | 9 (50) |
| Immunosuppressants | 6 (33%) | | Intravitreal Iluvien | 2 (11) |
| Biological agents | 3 (17%) | | Intravitreal Anti-VEGF (bevacizumab) | 1 (6) |
| **Other ocular history** | | | | |
| Glaucoma | 4 (22%) | | Glaucoma surgery | 3 (17) |
| Cataract surgery | 1 (6%) | | ERM | 4 (22) |
| VR Surgery | 4 (22%) | | Others | 2 (11) |

VR: Vitro-retinal ERM: Epiretinal membrane IVI: Intravitreal injection UMO: Uveitic macular oedema
IVMP: Intravenous methylprednisolone IMMP: Intramuscular methylprednisolone Anti VEGF
Immunosuppressants include Mycophenolate mofetil Biological agents include Ipilimumab and Adalimumab
