# Supplementary material for: Outcomes important to patients with non-infectious posterior segment-involving uveitis: a qualitative study
Source: BMJ Open Ophthalmol. 2020 Jul 21;5(1):e000481. doi: 10.1136/bmjophth-2020-000481 (PMC7375431; doi:10.1136/bmjophth-2020-000481)
Supplement: Supplementary data [file bmjophth-2020-000481supp003.pdf]

Supplementary Table 1: Carer characteristics

Characteristic		N (%)
**Gender**	Male	6 (60)
	Female	4 (40)
**Age range (yrs.)**	18-30	1 (10)
	31-45	1 (10)
	46-60	5 (50)
	Over60	3 (30)
**Ethnic group**	White	9 (90)
	Asian/Asian British	0 (0)
	Mixed/ multiple ethnic groups	0 (0)
	Black/African/Caribbean/Black British	1 (10)
	Other	0 (0)
**Relationship**	Spouse/Partner	7 (70)
	Mother	2 (20)
	Daughter	1 (10)
	Friend	0 (0)
**Duration of care**	1-4 years	4 (40)
	5-9 years	2 (20)
	10-15 years	0 (0)
	Over 15 years	4 (40)
**Carer living with patient**	Yes	8(80)
	No	2 (20)
